# Enhancing safety in CT-guided lung biopsies: correlation of MinIP imaging with pneumothorax risk prediction

**DOI:** 10.1186/s13244-024-01890-7

**Published:** 2025-01-13

**Authors:** Michael P. Brönnimann, Leonie Manser, Bernhard Gebauer, Timo A. Auer, Dirk Schnapauff, Federico Collettini, Alexander Pöllinger, Alois Komarek, Miltiadis E. Krokidis, Johannes T. Heverhagen

**Affiliations:** 1 Department of Diagnostic, Interventional and Paediatric Radiology, Inselspital, Bern University Hospital, University of Bern, Bern, Switzerland; 2 Department of Radiology, Charité—Universitätsmedizin Berlin, Berlin, Germany; 3 Clinician Scientist Program, Berlin Institute of Health at Charité-Universitätsmedizin Berlin, Berlin, Germany; 4 1st Department of Radiology, School of Medicine, National and Kapodistrian University of Athens, Areteion Hospital, Athens, Greece

**Keywords:** Pneumothorax, Hemorrhage, Biopsy, Risk factors, Tomography

## Abstract

**Objectives:**

This study aimed to evaluate whether minimum-intensity projection (MinIP) images could predict complications in CT-guided lung biopsies.

**Methods:**

We retrospectively analyzed 72 procedures from January 2019 to December 2023, categorizing patients by pneumothorax and the severity of hemorrhage (grade 2 or higher). Radiodensity measurements were performed using lung window (LW) and MinIP (10-mm slab) images. Regions of interest (ROIs) were placed at sites of the lowest density along the biopsy pathway. Absolute values were recorded, categorized by a radiodensity level of −850 HU, and assessed using our bridged radiological observations with measurement-optimized model (BROM-OLB) model with validation from three additional ROIs. Emphysema was visually scored. Statistical analysis included univariate analysis (Fisher’s exact and Mann–Whitney *U*-tests) and binomial logistic regression to identify confounders.

**Results:**

Lower radiodensity values in MinIP images in the access route, particularly with the BROM-OLB MinIP method, were significantly associated with a higher risk of pneumothorax (5/39, 13% vs 27/33, 82%, *p* < 0.01; Sensitivity 81.8% and Specificity 87.2%). Pneumothorax was more common with longer procedures (*p* < 0.05). Lower LW density values correlated with higher pulmonary hemorrhage rates (*p* < 0.01). Binomial logistic regression identified positive BROM-OLB MinIP results (OR 28.244, 95% CI: 7.675–103.9, *p* < 0.01) and lower LW density (OR 0.992, 95% CI: 0.985–0.999, *p* = 0.025) as independent risk factors. The optimal threshold values to predict pneumothorax were −868 HU in MinIP images and −769 HU in LW.

**Conclusion:**

The assessment of MinIP images is superior, and in combination with relative quantitative measurement of radiodensity for access route planning, it can reduce the risk of pneumothorax in CT-guided lung biopsies.

**Critical relevance statement:**

This article critically evaluates the risk factors for complications in CT-guided lung biopsies, highlighting the potential of MinIP images for predicting pneumothorax risk, thereby advancing clinical radiology practices to improve patient safety and reduce healthcare costs.

**Key Points:**

- This work investigates if MinIP images efficiently predict CT-guided lung biopsy complications.
- MinIP imaging identified higher pneumothorax risk post-CT lung biopsy with superior accuracy.
- Our method detects high-risk lung changes linked to pneumothorax without additional software.

**Graphical Abstract:**

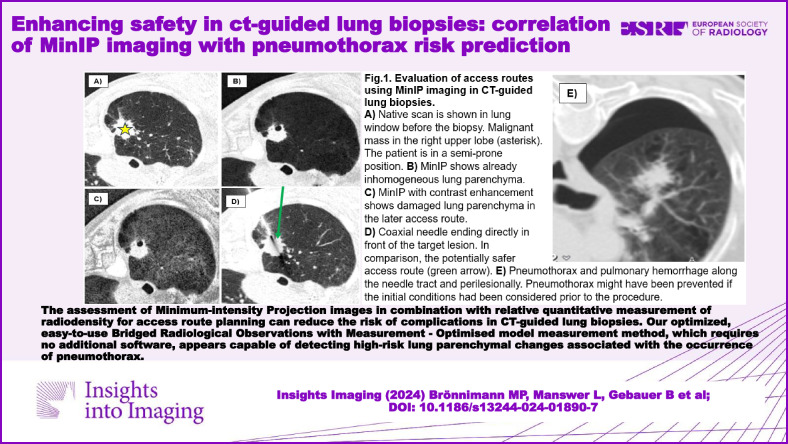

## Introduction

Pneumothorax is a common complication of computed tomography (CT)-guided lung biopsies, occurring in 8–69% of cases [1–14]. This complication occurs when air disrupts the pleural pressure that normally keeps the lung inflated [1, 7, 14]. While most pneumothoraces are small and resolve on their own, larger ones and associated pulmonary hemorrhages, which can occur in up to 20.9% of cases, often require additional treatment, significantly increasing healthcare costs and risks [6, 12, 15–25].

Patients over 65, who frequently undergo these biopsies, have a higher risk of pneumothorax due to reduced lung elasticity and common conditions like chronic obstructive pulmonary disease (COPD) [26–29]. Emphysema, a severe form of COPD, involves irreversible airspace enlargement and airway wall destruction, which increases the risk of complications [19, 27]. Visual and software-based methods were used to investigate the link between emphysema prevalence and pneumothorax occurrence, following earlier promising findings [19, 30–35].

Interestingly, inconsistent findings concerning pneumothorax risk have been reported to date. Theilig et al [36] noted, that quantitatively determined pulmonary emphysema is a positive predictor of the pneumothorax rate in CT‑guided lung biopsy. Using the visual method, Lee et al [19] reported that perilesional emphysema was significantly associated with pneumothorax (*p* < 0.001) and hemorrhage (*p* = 0.001). However, the severity of visual and quantitative emphysema was not a significant risk factor for pneumothorax or hemorrhage (*p* > 0.05) [19]. Zhang et al [37] reported only the lowest value of lung radiodensity along the needle passage was a quantitative predictor of pneumothorax. A value of −850 HU or less was an independent risk factor for pneumothorax. However, Asai et al [4] found no significant differences in COPD staging or low attenuation area scores between the patients with and without pneumothorax.

Possible explanations could include the inhomogeneous distribution of lung destruction and earlier or other parenchymal damage [38]. A potential solution could involve using post-processed minimum-intensity projection (MinIP) images, which project the voxel with the lowest attenuation value. This technique allows for prompt diagnosis of cystic lung diseases and airway, vascular, or parenchymal disorders that manifest with hypoattenuation, mosaic attenuation, or air trapping [39].

**Fig. 1:**
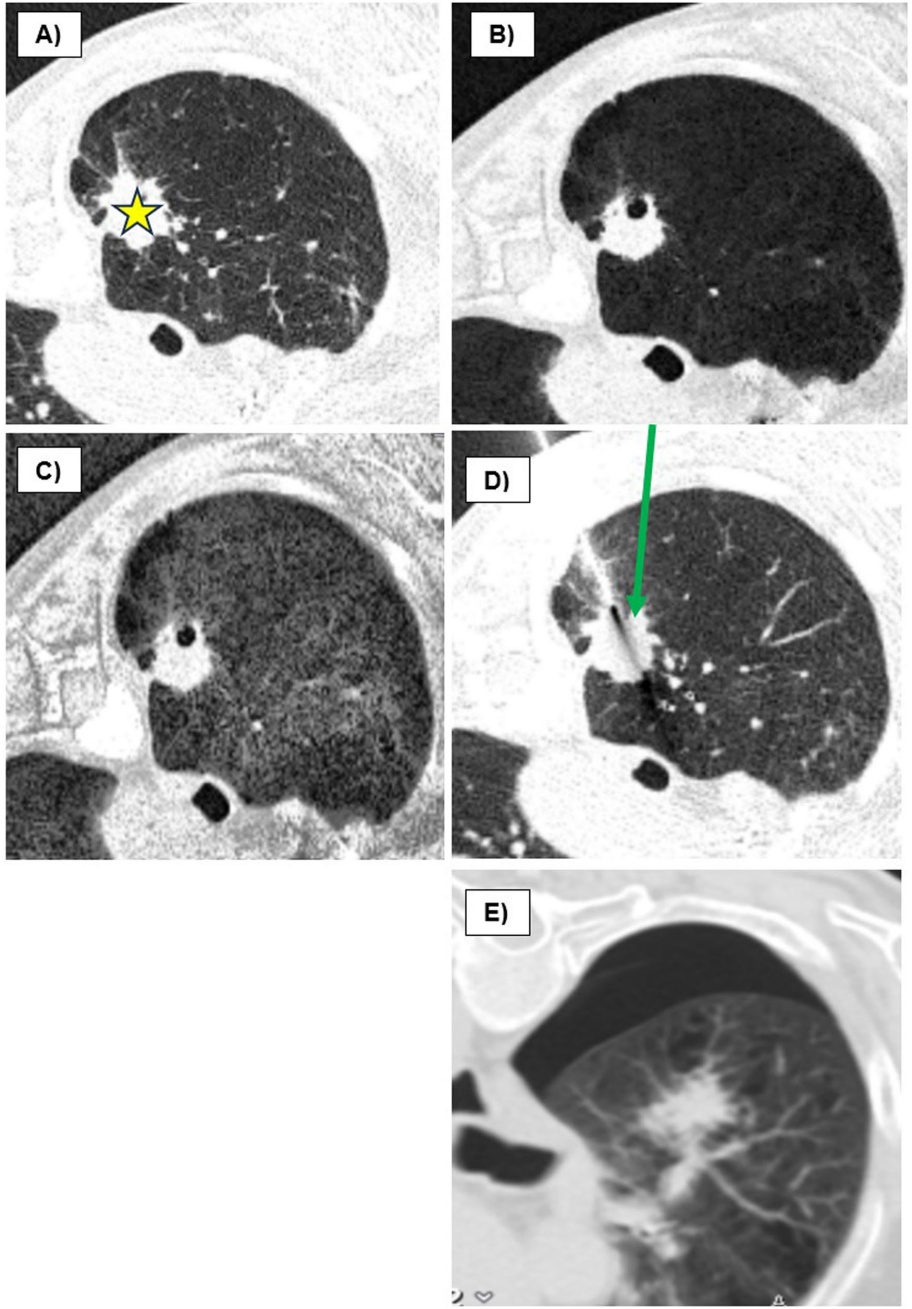
Evaluation of access routes using MinIP imaging in CT-guided lung biopsies. **A** Native scan is shown in the lung window (LW) before the biopsy. Malignant mass in the right upper lobe (asterisk). The patient is in a semi-prone position. **B** MinIP shows already inhomogeneous lung parenchyma. **C** MinIP with contrast enhancement shows damaged lung parenchyma in the later access route. **D** Coaxial needle ending directly in front of the target lesion. In comparison, the potentially safer access route (green arrow). **E** Pneumothorax and pulmonary hemorrhage along the needle tract and perilesionally. Pneumothorax might have been prevented if the initial conditions had been considered prior to the procedure

This study aimed to investigate whether the use and quantitative evaluation of MinIP images could predict complications from CT-guided lung biopsies (Fig. 1). The overall objective is to provide interventionalists with a potential tool to reduce the pneumothorax rate without the need for additional software.

## Methods

### Study population

**Fig. 2:**
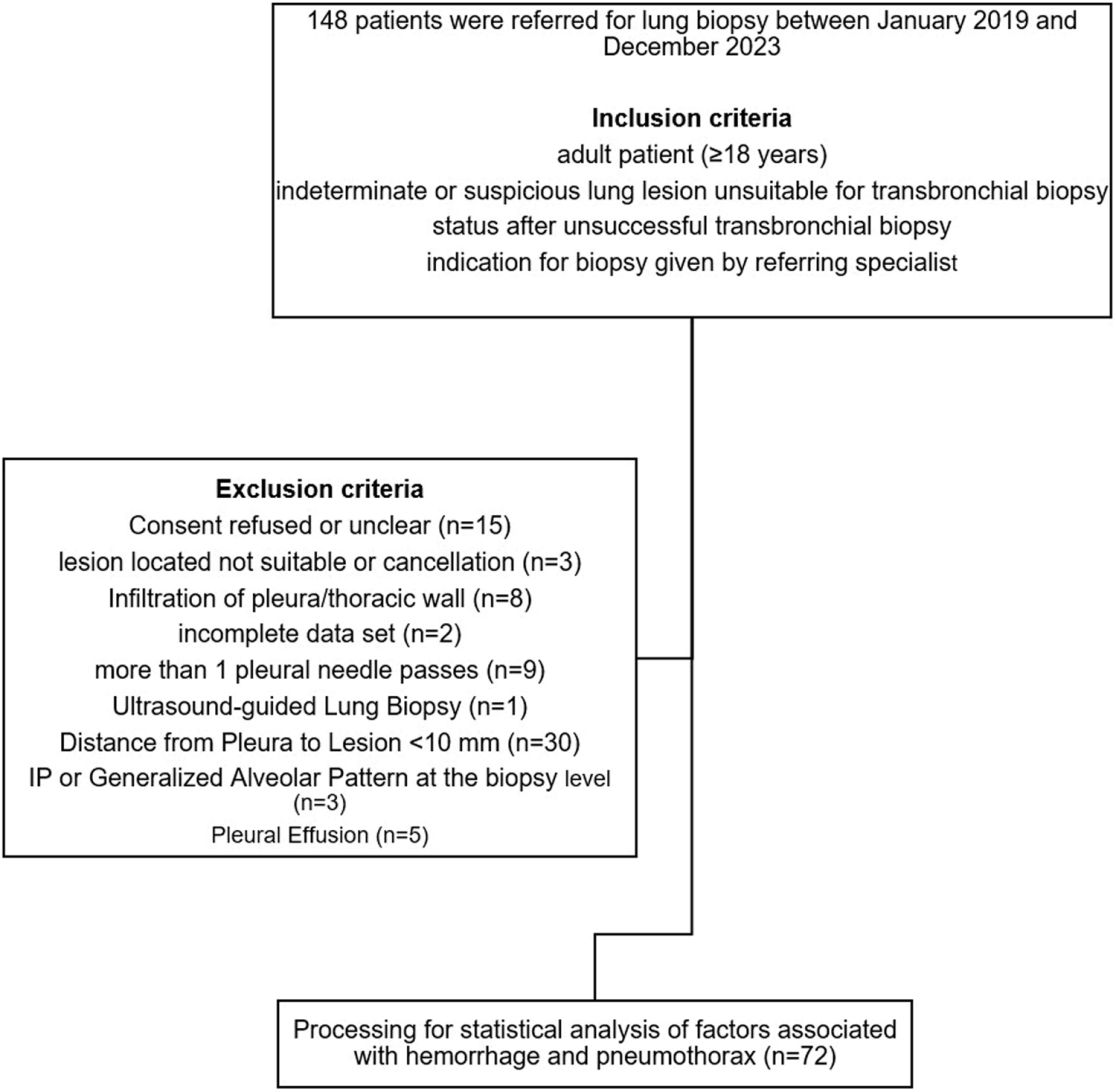
Flowchart shows the study population

This study conducted a retrospective analysis of 148 percutaneous CT-guided lung biopsies performed at our university hospital from January 2019 to December 2023. We defined a minimum distance of > 10 mm from the pleura to the target lesion for three key reasons. First, this distance enables accurate manual density measurements with minimal standard deviation. Second, it helps exclude inflammatory or infiltrative changes in the lung parenchyma caused by adjacent malignant lesions (safety margin, [40, 41]). Third, this margin facilitates the detection of potential peripheral air trapping [42]. Consecutive exclusion criteria were established to mitigate potential bias from predominant abnormal physiology in the pleural cavity or lung parenchyma (infiltration or effusion). Additionally, factors that could lead to false-positive results in the density measurement, such as interstitial pneumopathy or generalized alveolar patterns in the lung, were excluded. Finally, 72 cases met the inclusion criteria (Fig. 2).

### Baseline evaluation

All patients underwent a baseline clinical evaluation, including a medical history review and standard blood tests. Procedure requirements included an international normalized ratio value below 1.5 or a Quick value above 60%, a hemoglobin value exceeding 80 g/L, and a platelet count of over 50 × 10^9^/L, with blood values not older than 5 days. Non-steroidal anti-inflammatory drugs and clopidogrel were discontinued 5 days before the procedure, heparin 6 h before, rivaroxaban 1 day before, and dabigatran and endoxaban 3 days before, following established guidelines.

### Biopsy technique

If the biopsy posed a significant risk under normal breathing conditions, it was rescheduled to be performed under anesthesia. Four interventional radiologists, each with 7 to over 10 years of experience, conducted all lung biopsies. The procedures were CT-guided using a Toshiba Asteion 4SL scanner (and either a 17- or 19-gauge coaxial needle paired with an 18- or 20-gauge semiautomated biopsy system (0.828 pitch, 120-kVp tube energy, modulated tube current, and 0.5-s gantry rotation) SemiCut side-cutting only for 18-gauge; Medical Devices Lease S.A., Zug, Switzerland or CorVocetTM full-core; Merit Medical Systems, Utah, United States). Biopsy planning relied on a non-contrast chest CT with 1 mm reconstruction increments (512 × 512-pixel matrix, reconstruction kernel FC7), adhering to the gold standard for needle path planning. Special care was taken to avoid pulmonary vessels, and crossing pulmonary fissures and pleural effusion was forbidden. The interventionalist chose the patient’s positioning based on their experience and the patient’s capabilities. Local anesthesia (1% lidocaine, max 20 mL) was administered. Breathing instructions were omitted to prevent hyperventilation, which could have prolonged the procedure. After tissue sampling, the needle was promptly withdrawn without a sealing agent. A follow-up CT scan was performed immediately post-needle withdrawal, and if no complications arose, the patient was transferred to a supine position on a bed without relocating. If local hemorrhage occurred, another follow-up CT scan was conducted after 5 min. Coughing and hemoptysis were tolerated with stable vital signs, and patients applied counterpressure using the Valsalva maneuver while elevated. A drainage system (Safe-T-Centesis TM 6 or 8F) was utilized if progressive pneumothorax was detected. Patients were monitored for routine vital signs for 4 h post-procedure, and if asymptomatic with no complications, were discharged home. If the control CT scan revealed a non-progressive pneumothorax, a chest X-ray was performed 6 h later for further evaluation. If the pneumothorax exceeded 2 cm, the patient was admitted for one night.

### Data collection and image analysis

All procedures were reviewed by a board-certified interventional radiologist with eight years of experience and a radiology resident with three years of experience, both were blinded to the patient’s medical history and did not perform any interventions. All interventional images were analyzed with the software Sectra Workstation (Model IDS7, version 24.2, Patch 4/2022; Sectra AB, Linköping, Sweden).

### Radio density measurements

The planning scan reconstructed in lung window (LW) (1 mm reconstruction increments) with a width of 1500 and level of −500 served as the basis. Regions of interest (ROIs) with a diameter of approximately 5 mm are set within the lowest value alongside the access route serving as a reference. Five millimeters was chosen as experience has shown that the lateral error of coaxial needles is usually within this range. The values were recorded in the reconstructed LW and in the MinIP (slap thickness 10 mm) images. Following the findings of Zhang et al [37] we graded our results based on a radiodensity threshold of −850 Hounsfield Units (HU).

**Fig. 3:**
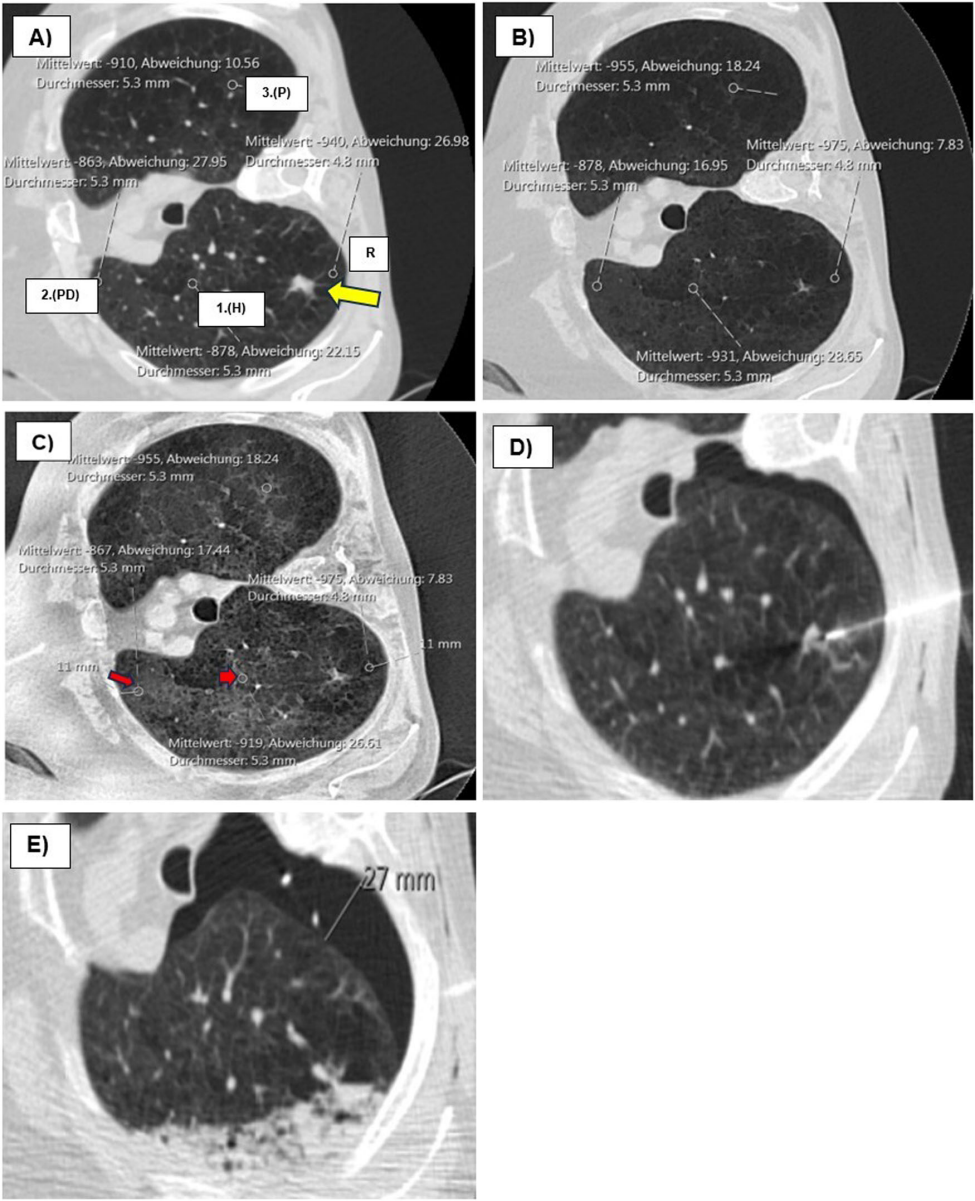
Technical realization: **A** native scan is shown in the LW: patient in lateral position and target lesion in the posterior upper lobe segment on the right (arrow). ROI with approx. Five millimeters, and the lowest value in the access route is taken as a reference (R). Three other ROIs of the same size are used for comparison: at the same height (1.H), at the same distance from the pleura (2.PD), and in the parenchyma (3.P) (each in the same or different lung lobe). It is essential to follow the specified order for setting additional ROIs within this model. Measurements within visually destructed lung parenchyma or ground glass opacity formations are prohibited. The deviation from the mean measurement value between the respective ROIs must not exceed 20 HU. Positive rating if the density of the ROI in the access route was lower than the majority of the other ROIs (the same rule also applies in the MinIP over the entire slap thickness). **B** The same ROIs in the MinIP (slap thickness 10 mm). **C** After MinIP CE adjustment of the marked ROI (red arrows—shift posteriorly) in visually inconspicuous parenchyma. 2. PD maintains the distance to the pleura from 11 mm. **D** Access the route with a coaxial needle in front of the target lesion. **E** Pneumothorax with a seam width of 27 mm and grade 3 lobar hemorrhage. Mittelwert, mean; Abweichung, deviation; Durchmesser, diameter; mm, millimeter

To reduce the variability of measured values and compensate for differences in X-ray beam energies [43] and location-related radiodensity variations [15, 44], we applied our Bridged Radiological Observations with a measurement-optimized model (BROM-OLB) for the relative quantitative measurement of radiodensity in CT-guided lung biopsies. The design of this model aimed to generate a reliable depiction of the heterogeneous lung parenchyma, accessible to any interventionalist before biopsy without requiring supplementary software. Three additional ROIs of identical diameter were positioned for comparison with the initial reference ROI, which was placed at the lowest density value along the access route. Their selection was guided by specific criteria to ensure consistency and address potential density variations. Given the height-dependent nature of lung density [45], the first additional ROI was placed at the same vertical level as the ROI along the access route. This positioning aimed to account for variations in density due to gravity and to improve the consistency and reliability of density measurements in different lung regions. The second additional ROI was positioned at an equivalent distance from the pleura, ideally at the same vertical level or within the same third of the lung as the target lesion, to account for density variations from the central to peripheral regions of the lung [46]. The third and final ROI was positioned within visually normal lung parenchyma, either within the same lobe or in a different lung lobe, to serve as a control reference for typical parenchymal density. If the visually normal lung parenchyma had already been measured by the first ROI at the same vertical level, the third ROI was positioned in the other lung. Because the vertical gradient has a more pronounced effect on lung density than the horizontal gradient, it was essential to follow the specified order for setting additional ROIs within this model. Measurements within visually distorted lung parenchyma or ground glass opacity formations were prohibited. This restriction helped to reduce the risk of interference from pathological alterations that could bias density measurements. For our ROI measurements, we selected 1 mm reconstructed images instead of the 5-mm slices commonly used in other studies. This higher-resolution approach provided enhanced spatial detail, allowing for more precise differentiation of small density variations. By reducing the partial volume effect, 1-mm slices offer improved accuracy in capturing subtle density changes across ROI, thereby ensuring the precision necessary for detailed analysis. Additionally, the finer resolution of 1-mm slices enables the accurate positioning of ROIs within clear, homogeneous lung regions, minimizing the risk of artifacts or mixed tissue influences that could otherwise distort density values. To further enhance reliability, we restricted the allowable deviation from the mean measurement value between ROIs to within 20 HU, helping to control for potential attenuation errors while using high-resolution images. A positive rating was assigned if the density of the ROI within the access route was lower than that of most of the other ROIs (the same criterion applied to the minimum intensity projection (MinIP) across the entire slab thickness). In the second step, a MinIP with a slab thickness of 10 mm was generated. Edge sharpening (35%) and contrast enhancement (20%) were added to the MinIP. The ROIs were then re-evaluated and, if necessary, repositioned in more normal lung parenchyma (Fig. 3).

### Emphysema, pneumothorax, pulmonary hemorrhage grading, and other investigated parameters

**Fig. 4:**
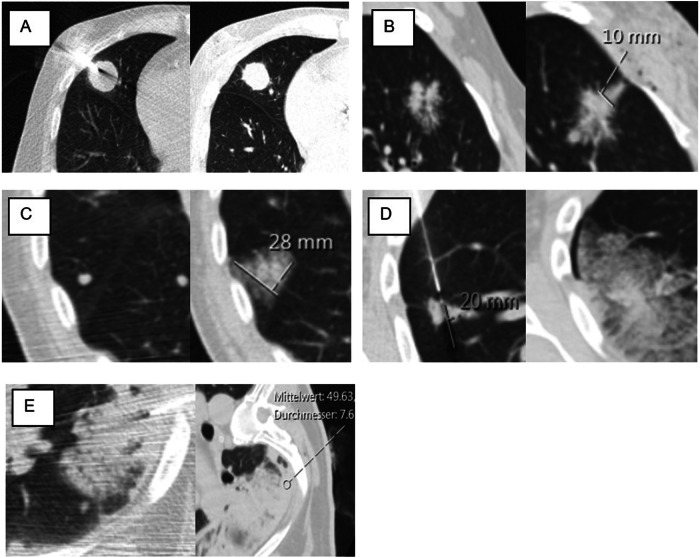
Pulmonary hemorrhage grading system. CT scan performed before/during (left) and directly after (right) demonstrate the following grades **A** Grade 0 without image morphological evidence of parenchymal hemorrhage after biopsy. **B** Grade 1: new linear, fine ground glass with a width under 2 cm can be delineated in the access route after the biopsy. **C** Grade 2: after the biopsy, the target lesion can no longer be demarcated if the alveolar hemorrhage is locally larger than 2 cm. **D** Grade 3: lobar hemorrhage and the biopsy channel before. **E** Grade 4: hemaothorax with immediate hyperdense fluid within the pleural cavity after biopsy. Mittelwert, mean; Durchmesser, diameter; mm, millimeter

The approved visual assessment system for grading emphysema, according to the Fleischner Society, was used and consequently only recorded in the reconstructed LW images [30]. Binary evaluation of the presence of a pneumothorax on the immediate post-biopsy CT control images, regardless of severity. Pulmonary hemorrhage was evaluated based on the appearance of new consolidative or ground-glass opacity on post-biopsy images and classified using a consensus-based grading system adapted from previous research [16, 18, 47]: Grade 0 indicated no pulmonary hemorrhage, Grade 1 represented needle tract hemorrhage ≤ 2 cm (Fig. 4A, B), Grade 2 indicated hemorrhage > 2 cm but confined to sub lobar regions (Fig. 4C), Grade 3 denoted lobar hemorrhage or larger (Fig. 4D), and Grade 4 indicated hemothorax (Fig. 4E). Other variables assessed included intervention date, birth date, age, sex, procedure time (in minutes), patient position, lesion size (in mm), lesion location, biopsy angle, distance from skin to the lesion (in mm), distance from pleura to lesion (in mm), and lesion location. Needle size, needle system, and the number of samples were recorded from the intervention report. Histological results from the target lesion and the patient’s post-interventional history were collected retrospectively from electronic medical records.

### Statistical analysis

Statistical analyses were performed using commercially available software (IBM SPSS Statistics for Windows, version 28; IBM, Armonk, NY). A Chi-Square and Fisher exact tests for categorical variables, and the Mann–Whitney *U*-test for continuous variables. The Kolmogorov-Smirnov test assessed a normal distribution. The Spearman correlation for continuous and contingency correlation for categorical values was used to identify high correlations between the variables. The phi coefficient was calculated for categorical variables and the Pearson correlation coefficient for continuous variables to determine effect sizes. In cases of highly correlated variables, only the variable with the highest effect size was included in the logistic regression model. The optimal measuring method should demonstrate high sensitivity and area under the curve (AUC) while maintaining a low number of false negatives. To evaluate the optimal threshold from the ROC analysis, the Youden Index and the point closest to the top left were used. All tests were performed two-sided with a level of significance *p* < 0.05. As the side-cutting biopsy system is only available for 18-gauge size (SemiCut, Medical Devices Lease S.A., Zug, Switzerland), homogenization had to be performed for direct comparison of the groups (needle size and system), and cases had to be excluded. To avoid overfitting the regression model, we adhered to the rule of ten [48]. As a result, we included three independent variables in the logistic regression model based on the number of events in the smallest outcome category. For this purpose, we selected the variables that were significant or close to significance, considering a minimum group size of *n* ≥ 25 for categorical predictors. A logistic regression model was used to assess potential risk factors for pneumothorax and pulmonary hemorrhage [49].

## Results

### Study population

The patients analyzed were, on average, 67.46 ± 11.40-years-old, and the gender distribution (47% women) was almost equally distributed. A total of 79% of the biopsied lung nodules were malignant, with the majority being metastases and approximately one-third being primary lung tumors. Just over 20% of the nodules were benign lesions (Fig. S1). Age, sex, lesion size, lesion location, needle system (18 G), number of samples, biopsy angle, the skin-to-lesion, pleura-to-lesion distance, and subgroups of the general emphysema and emphysema in the access route did not differ significantly between patients (Tables 1A, B and 2A, B). The analyzed variables were not normally distributed in the groups.

**Table 1:** Univariate analysis of patient demographics and interventional parameters

(A) Survey of lung biopsies										
Parameter	All (*n* = 72)		No pneumothorax (*n* = 39)		Pneumothorax (*n* = 33)		*p* value	φ	CC/SC	PC
Female	34	47%	18	46%	16	48%	1.000			
Age, (years)	67.46	±11.40	67	±12.48	68.83	±9.556	1.000			
Lesion size, (mm)	20.51	±9.80	21.74	±11.76	19.06	±6.73	0.534			
Patient position							0.153			
Supine	18	25%	7	18%	11	33%				
Lateral	29	40%	15	38%	14	42%				
Prone	25	35%	17	44%	8	24%				
RQM of radio density (*n*)										
BROM-OLB MinIP positive	32	44%	5	13%	27	82%	< 0.001^∗^	0.692		
MinIP −850 HU	42	58%	14	36%	28	85%	< 0.001^∗^	0.495	< 0.001^∗^	
MinIP value	−866.9	±132.78	−817	±142.54	−926	±91.339	< 0.001^∗^		0.041^∗^	−0.402
BROM-OLB LW positive	29	40%	5	13%	24	73%	< 0.001^∗^	0.609	< 0.001^∗^	
LW −850 HU	12	17%	5	13%	7	21%	< 0.001^∗^	0.411	< 0.001^∗^	
LW value	−745.9	±130.49	−699.9	±143.795	−800.36	±87.113	< 0.001^∗^		0.973	−0.469
Needle size (full-core)							0.068			
18 G	27	63%	9	28%	7	64%				
20 G	16	37%	23	72%	4	36%				
Biopsy system (18 G)							0.051			
Side-cut	29	40%	7	18%	22	67%				
Full-core	16	22%	9	23%	7	21%				
Number of samples (*n*)							0.985			
1 and 2	16	22%	8	21%	8	24%				
3	33	46%	18	46%	15	45%				
4	18	25%	10	26%	8	24%				
5	5	7%	3	8%	2	6%				

(B) Survey of lung biopsies									
Parameter	All (*n* = 72)		No pneumothorax (*n* = 39)		Pneumothorax (*n* = 33)		*p* value	φ	CC
Biopsy angle (degree)	61.64	±18.84	60.82	±19.87	62.61	±17.79	0.747		
Distance SL, (mm)	68.63	±19.15	70.00	±20.72	67	±17.29	0.667		
Distance PL, (mm)	24.99	±13.70	23.23	±11.82	27.06	±15.58	0.428		
Lesion location							0.285		
UL	35	49%	16	41%	19	58%			
LL	32	44%	19	49%	13	39%			
ML/L	5	7%	4	10%	1	3%			
General emphysema	29	40%	9	23%	20	61%	0.002^∗^	0.381	< 0.001^∗^
General FSVE							0.214		
None	9	13%	4	10%	5	15%			
Mild	7	10%	3	8%	4	12%			
Moderate	10	14%	4	10%	6	18%			
Confluent	11	15%	1	3%	10	30%			
Advanced destruction	1	1%	1	3%	0	0%			
Emphysema LAR	22	31%	6	15%	16	48%	0.004^∗^	0.358	< 0.001^∗^
LAR E FSVE							0.601		
None	15	21%	7	18%	8	24%			
Mild	5	7%	1	3%	4	12%			
Moderate	10	14%	3	8%	7	21%			
Confluent	8	11%	2	5%	6	18%			
Advanced destruction	0	0%	0	0%	0	0%			
Procedure time, (min)	27.72	±8.46	25.95	±7.89	29.82	±8.75	0.041^∗^		

Unless stated otherwise, data are mean ± standard deviation. X^2^ (R X 2), Fisher’s exact test, and the Mann–Whitney *U*-test were used to calculate the statistical difference between groups of categorical, dichotomous, and continuous variables, respectively

Testing correlation for categorical (CC) and continuous (SC) variables, with effect size comparison for categorical variables (phi coefficient, φ) and for continuous variables (PC)

*Y* year, *mm* millimeter, *RQM* relative quantitative measurement of radio density, *BROM-OLB* optimized model for the relative quantitative measurement of radio density in CT-guided lung biopsies, *MinIP* minimum intensity projection, *LW* lung window, *HU* Hounsfield units, *G* gauge, φ phi-coefficient, *CC* contingency coefficient, *SC* Spearman correlation, *PC* Pearson correlation coefficient, *SL* skin to lesion, *PL* pleural to lesion, *UL* upper lobe, *LL* lower lobe, *ML* middle lobe, *L* lingula, *FSVE* Fleischner society visual emphysema CT pattern, *LAR E* emphysema at the level of access route

^∗^ Statistically significant (defined *p* < 0.05). Testing correlation for categorical (CC) and continuous (SC) variables, with effect size comparison for categorical variables (phi coefficient, φ) and for continuous variables (PC)

**Table 2:** Univariate analysis for the evaluation of pulmonary hemorrhage

(A) Survey of lung biopsies								
Parameter	All (*n* = 72)		None or Grade 1 hemorrhage (*n* = 52)		Grade 2 or higher hemorrhage (*n* = 20)		*p* value	PC
Female	34	47%	24	46%	10	50%	1.000	
Age, (years)	67.46	±11.40	67.44	±12.393	67.5	±8.593	0.585	
Lesion size, (mm)	20.51	±9.80	20.42	±10.007	20.75	±9.525	0.660	
Patient position							0.153	
Supine	18	25%	7	13%	11	55%		
LD	29	40%	15	29%	14	70%		
Prone	25	35%	17	33%	8	40%		
RQM of radio density (*n*)								
BROM-OLB MinIP positive	32	44%	22	42%	10	50%	0.604	
MinIP −850 HU	42	58%	30	58%	12	60%	0.787	
MinIP value	−867	±132.78	−862.98	±146.347	−878	±86.495	0.759	
BROM-OLB LW positive	29	40%	20	38%	9	45%	0.789	
LW −850 HU	12	17%	8	15%	4	20%	0.727	
LW value	−746	±130.49	−725.04	±142.635	−804.26	±59.285	0.027^∗^	−0.259
Needle size (full-core)							0.209	
18 G	16	37%	8	30%	8	50%		
20 G	27	63%	19	70%	8	50%		
Biopsy system (18 G)							1.000	
Side-cut	29	40%	21	40%	8	40%		
Full-core	16	22%	31	60%	12	60%		
Number of samples (*n*)							0.340	
1 and 2	16	22%	14	27%	2	10%		
3	33	46%	21	40%	12	60%		
4	18	25%	13	25%	5	25%		
5	5	7%	4	8%	1	5%		

(B) Survey of lung biopsies							
Parameter	All (*n* = 72)		None or Grade 1 hemorrhage (*n* = 52)		Grade 2 or higher hemorrhage (*n* = 20)		*p* value
Biopsy angle (degree)	61.64	±18.84	61.12	±19.11	63	±18.52	0.743
Distance SL, (mm)	68.63	±19.15	69.35	±19.251	66.75	±19.265	0.415
Distance PL, (mm)	24.99	±13.70	24.6	±12.83	26	±16.09	0.983
Lesion location							0.845
UL	35	49%	24	46%	11	55%	
LL	32	44%	24	46%	8	40%	
ML/L	5	7%	4	8%	1	5%	
General emphysema	29	40%	20	38%	9	45%	0.789
General FSVE							0.319
None	9	13%	4	10%	5	15%	
Mild	7	10%	4	10%	3	9%	
Moderate	10	14%	8	21%	2	6%	
Confluent	11	15%	8	21%	3	9%	
Advanced destruction	1	1%	0	0%	1	3%	
Emphysema LAR	22	31%	15	29%	7	35%	0.776
LAR E FSVE							0.574
None	15	21%	8	21%	7	21%	
Mild	5	7%	3	8%	2	6%	
Moderate	10	14%	8	21%	2	6%	
Confluent	8	11%	4	10%	3	9%	
Advanced destruction	0	0%	0	0%	0	0%	
Procedure time (min)	27.72	±8.46	25.95	±7.89	29.82	±8.75	0.353

Unless stated otherwise, data are mean ± standard deviation. X^2^ (R X 2), Fisher’s exact test, and the Mann–Whitney *U*-test were used to calculate the statistical difference between groups of categorical, dichotomous, and continuous variables, respectively

*Y* year, *mm* millimeter, *RQM* relative quantitative measurement of radio density, *BROM-OLB* optimized model for the relative quantitative measurement of radio density in CT-guided lung biopsies, *MinIP* minimum intensity projection, *LW* lung window, *HU* Hounsfield units, *G* Gauge, *PC* Pearson correlation coefficient, *SL* skin to lesion, *PL* pleural to lesion, *UL* upper lobe, *LL* lower lobe, *ML* middle lobe, *L* Lingula, *FSVE* Fleischner society visual emphysema CT pattern, *LAR E* emphysema at the level of access route

^*^ Statistically significant (defined *p* < 0.05). Effect size comparison for continuous variables (PC)

### Complications after CT-guided lung biopsy

Forty-five point eight percent was the pneumothorax rate, 12.5% a drainage had to be inserted, 27.7% pulmonary hemorrhage (grade 2 or higher) resulted, and the success rate in terms of obtaining a positive histopathological result was 94.4%. 70% of pneumothorax occurred in the age group of 70–84, and 36% of pulmonary hemorrhage (grade 2 or higher) in the age group of 55–69 (Fig. S2). Confluent emphysema in the assessment, according to Fleischner Society visual emphysema CT pattern (FSVE), was in general (15%) and in the group with pneumothorax (30%) the most common. Two pulmonary hemorrhages resulted in grade 3 (3%) and one in grade 4 (1%). One of these required admission and monitoring. None required further intervention or transfusion. In 1% of the patients, alveolar hemorrhage was not even detectable in the access route (grade 0). The univariate analysis revealed that pneumothorax occurred significantly more frequently when the radiodensity was lower, regardless of the measuring method used or the measured values (*p* < 0.01). Additionally, pneumothorax was more common if general emphysema (*p* < 0.01) and emphysema at the level of the access route (*p* < 0.01) were present, and if the procedure lasted longer (*p* < 0.05) (Table 1A, B). Pulmonary hemorrhage (grade 2 or higher) occurred significantly more frequently when the measured density values in the reconstructed LW were lower overall (*p* < 0.01) (Table 2A, B). The BROM-OLB MinIP positivity is strongly associated with the occurrence of pneumothorax across all subgroups, with statistically significant *p* values in each category. The test performs best in the emphysema LAR-positive subgroup, which has both high sensitivity and perfect specificity. The test is slightly less effective in the inconspicuous subgroup (neither general nor LAR emphysema), with reduced sensitivity but still high specificity (Table 3).

**Table 3:** Univariate subgroup analysis for patients with general emphysema, emphysema LAR, and neither

	Total = 72		No pneumothorax			Pneumothorax			*p* value
	**General emphysema positive** ***n*** = **29**		***n*** = **9**			***n*** = **20**			
BROM-OLB MinIP positive	20	28%	2	22%		18	90%	Sens 90%	< 0.001^∗^
BROM-OLB MinIP negative	9	13%	7	78%	Spez 77.8%	2	10%		
	**Emphysema LAR positive** ***n*** = **22**		***n*** = **6**			***n*** = **16**			
BROM-OLB MinIP positive	18	25%	2	33%		16	100%	Sens 100%	0.002^∗^
BROM-OLB MinIP negative	4	6%	4	67%	Spez 66.67%	0	0		
	**No general or LAR emphysema** ***n*** = **43**		***n*** = **30**			***n*** = **13**			
BROM-OLB MinIP positive	12	17%	3	10%		9	69%	Sens 69%	< 0.001^∗^
BROM-OLB MinIP negative	31	43%	27	90%	Spez 90%	4	31%		

^∗^ Statistically significant (defined *p* < 0.05)

*BROM-OLB* optimized model for the relative quantitative measurement of radio density in CT-guided lung biopsies, *LAR* at the level of the access route

### Association of lesion characteristics, picture analysis, and interventional parameters with the occurrence of pneumothorax

The Spearman and contingency correlation analysis revealed that the image analysis results were highly correlated. Consequently, using the phi coefficient as an expression of the effect size, it was decided to include only BROM-OLB MinIP positive in the pneumothorax logistic regression model and LW Value in the pulmonary hemorrhage regression model. Binomial logistic regression analysis showed that positive BROM-OLB MinIP assessment (OR 28.244, 95% CI: 7.675–103.9, *p* < 0.01) was independently associated with a higher incidence of pneumothorax (Table 4). A good model fit with an *R*² = 0.55, *p* < 0.01. Cohen’s *f*^2^ was 1.22, corresponding to a strong effect [50]. Lower density values in LW were also independently associated with a higher incidence of pulmonary hemorrhage (grade 2 or higher) (OR 0.992, 95% CI: 0.985–0.999, *p* = 0.025) (Table 5). A modest model fit with an *R*² = 0.140, *p* < 0.01. Cohen’s *f*^2^ was 0.16, corresponding to a medium effect for our second regression model [50]. For completeness, we performed the binomial logistic regression separately for the other highly correlated variables, but each time with a significantly worse model fit.

**Table 4:** Binomial logistic regression analysis for pneumothorax risk

Variable	*B*	S.E.	Wald test	d*f*	*p* value	Odds ratio	95% CI	
							−	+
BROM-OLB MinIP positive	3.341	0.665	25.256	1	< 0.001^∗^	28.244	7.675	103.9
Procedure time, (min)	0.025	0.04	0.404	1	0.525	1.026	0.948	1.109

^∗^ Statistically significant (defined *p* < 0.05)

The total number of cases in the cohort for the multivariable analysis was *n* = 72

*B* regression coefficient (log odds), *S.E.* standard error—precision of the coefficient estimate; the Wald test statistic evaluates the null hypothesis that the coefficient is zero, indicating no effect, d*f* the degrees of freedom for the Wald test, *CI* confidence interval, *MinIP* minimum intensity projection, *min* minutes

**Table 5:** Binomial logistic regression analysis for Grade 2 or higher pulmonary hemorrhage

Variable	*B*	S.E.	Wald test	d*f*	*p* value	Odds ratio	95% CI	
							−	+
LW value	−0.008	0.004	5.048	1	0.025^∗^	0.992	0.985	0.999

^∗^ Statistically significant (defined *p* < 0.05)

The total number of cases in the cohort for the multivariable analysis was *n* = 72

*B* regression coefficient (log odds), *S.E.* standard error—precision of the coefficient estimate; the Wald test statistic evaluates the null hypothesis that the coefficient is zero, indicating no effect, d*f* the degrees of freedom for the Wald test, *CI* confidence interval, *LW* lung window

**Fig. 5:**
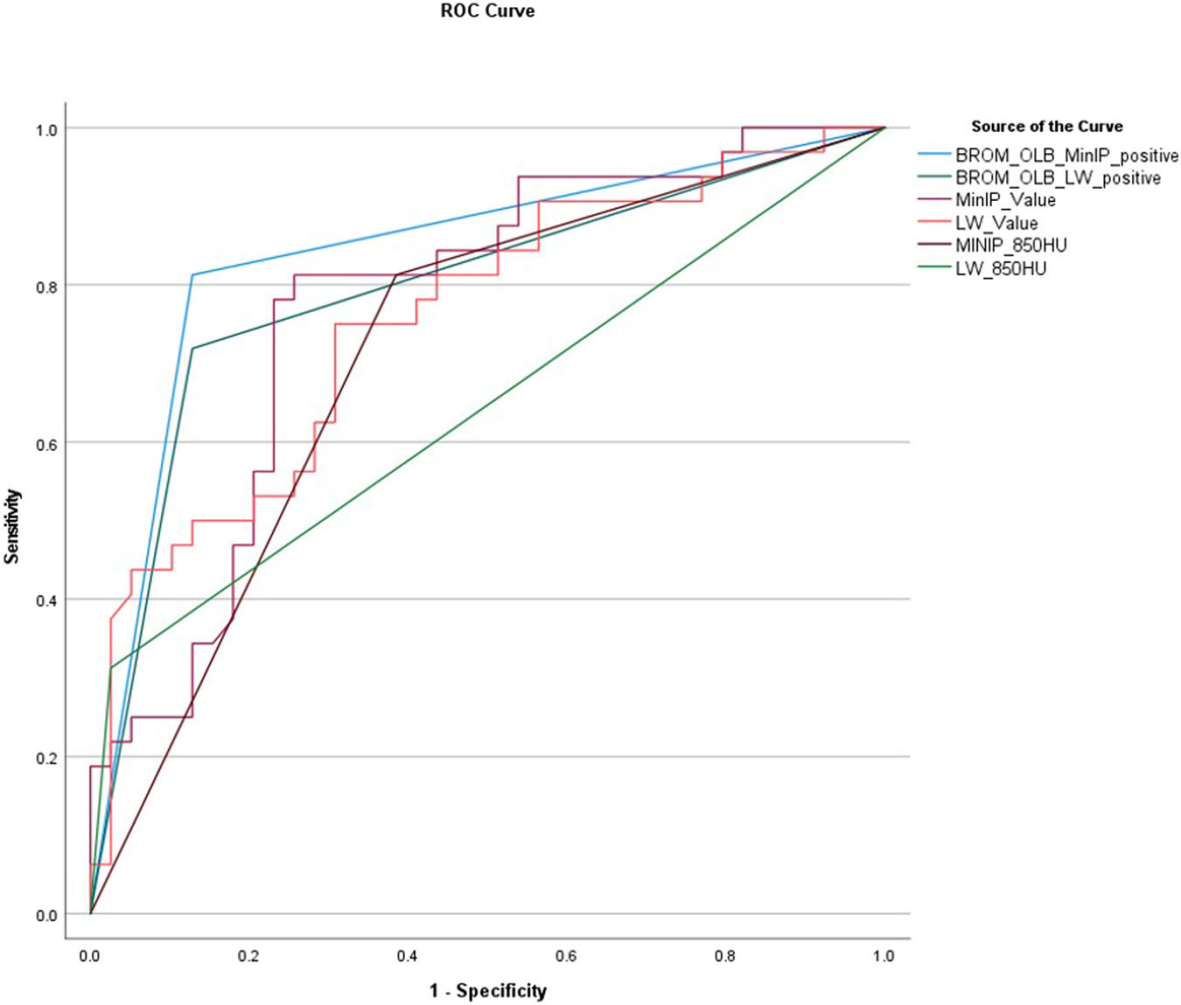
Receiver operating curve (ROC) of measuring methods for pneumothorax. BROM-OLB, optimized model for the relative quantitative measurement of radio density in CT-guided lung biopsies. MinIP, minimum intensity projection; LW, lung window; HU, Hounsfield units; G, general; LAR E, emphysema at the level of access route

Performance metrics of measurement methods to predict pneumothorax showed that BROM-OLB MinIP provided the best results (AUC 0.845, sensitivity 81.8%, and specificity 87.2%) and BROM-OLB, regardless of which image was measured, was superior to the other measurement methods. Regardless of the measuring method, the use of MinIP particularly increased sensitivity (81.8%) and reduced false-negative predictions from 9 to 6 (BROM OLB MinIP vs LW) and from 22 to 6 (MinIP −850 HU vs LW −850 HU), respectively. The optimum threshold value from ROC to predict pneumothorax was −868 HU in the MinIP and −769 HU in the LW (Fig. 5 and Table S1).

## Discussion

**Fig. 6:**
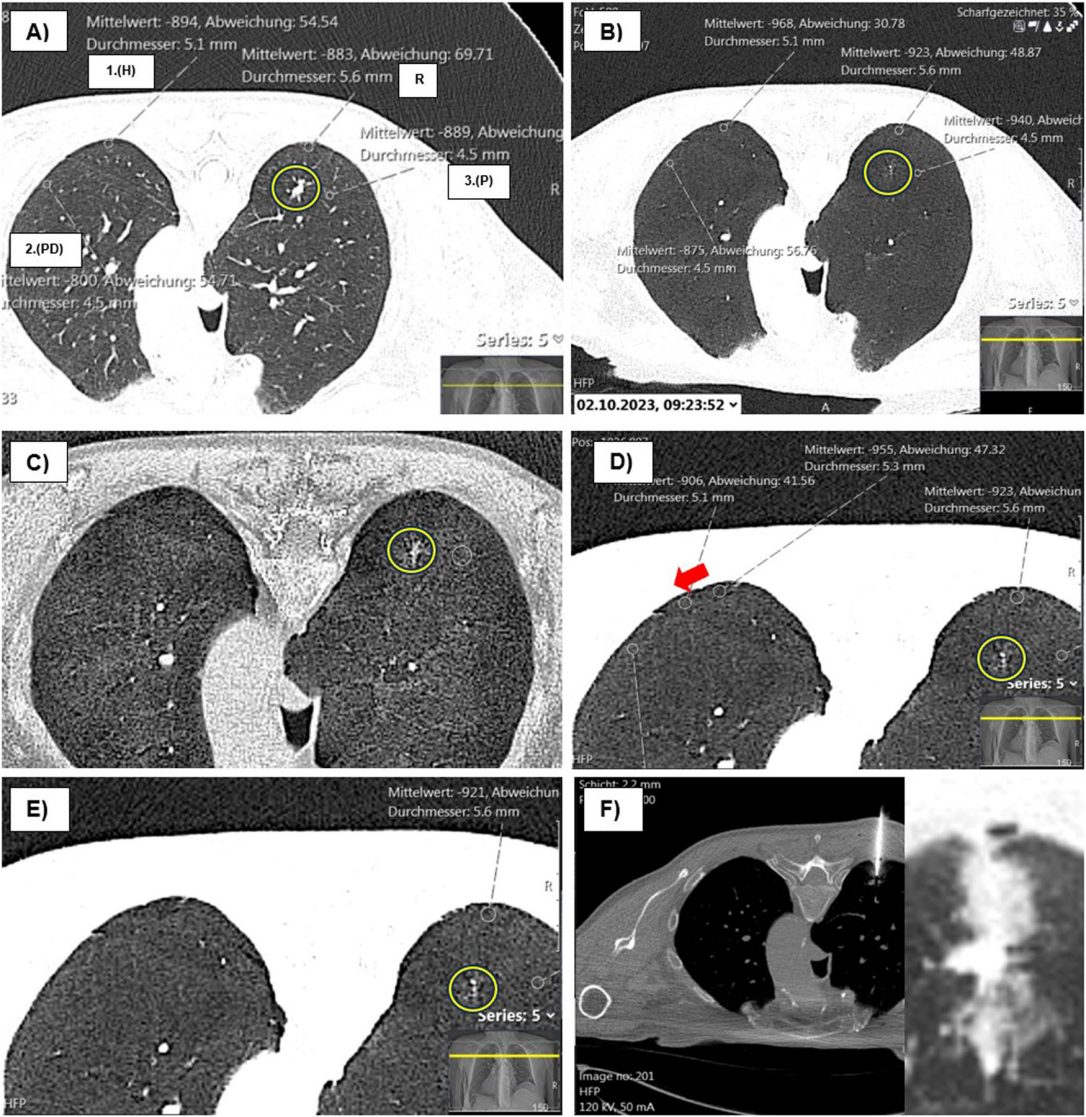
Superiority of the MinIP over the LW. **A** Target lesion (yellow circle) in the posterior upper lobe segment on the left and the patient in the prone position. A relatively unremarkable lung parenchyma is visually seen in the LW, which is also suggested by the initial measurements. **B** MinIP and, in particular, the MinIP CE (**C**), as well as the corresponding measurements, suggest possible hyperinflation or localized lung parenchymal damage. **D** Consequently, the ROI was shifted laterally (red arrow), and thus, the assessment in the MinIP was positive, contrary to that in the LW. **E** Left lung in which the ROI was measured at the same level and shifted with recognizable, locoregional parenchymal abnormality. **F** Biopsy of the target lesion with developing pneumothorax. Mittelwert, mean; Abweichung, deviation; Durchmesser, Diameter; mm, millimeter; R, reference ROI; 1.H, first additional ROI positioned at the same vertical level as the reference ROI; 2.PD, second additional ROI at an equivalent distance from the pleura as the reference ROI; and 3.P, third additional ROI placed within the lung parenchyma

Our study showed that when the radiodensity in the access path, as measured in the MinIP, was lower than the reference values in other areas of the unaffected lung parenchyma using our BROM-OLB measurement method, it was associated with a 28-fold increase in the risk of post-interventional pneumothorax (*p* < 0.01). Among the relative quantitative radiodensity measurement methods investigated, this method showed the best performance (AUC 0.845, sensitivity 81.8%, and specificity 87.2%). In general, however, the use of MinIP images for assessments is superior to those in the LW (Fig. 6). Higher radiodensity values in the access path, as measured in the LW, were associated with a risk reduction of 0.2% for pulmonary hemorrhage (grade 2 or higher) (*p* = 0.025). This is crucial, as the needle path can be strategically selected to avoid abnormal lung parenchyma in the access route, especially with the advent of new laser-guided CT systems that facilitate out-of-plane needle access. These user-friendly methods, which require no additional software, can be employed to significantly reduce the most common and serious complications of CT-guided lung biopsies.

The findings of this study corroborate and extend those of other research groups. We support the results of Lee et al [47] (visual method) and Zhang et al [37] (quantitative method), who both demonstrated that perilesional emphysema acts as an independent predictor for pneumothorax. Possible explanations for the inconsistent findings in the literature regarding the effect of generalized emphysema and its grading on the probability of pneumothorax occurrence can be derived from our results. For example, Theilig et al [36] demonstrated that quantitatively assessed pulmonary emphysema was a positive predictor of pneumothorax incidence, whereas many other authors using visual assessments could not show an association (e.g., Lee et al [47], Anderson et al [51], Yeow et al [18], Asai et al [4]). Thus, our results support the tendency that quantitative analysis is superior (quantitative measurement methods *p* < 0.01 vs visual assessment of general emphysema (*p* = 0.002) and at the level of the access route (LAR, *p* = 0.004), likely due to lack of inter-reader variability. Our additional results regarding the severity of emphysema also provide indirect support for this. Although the visual assessment showed no significant difference in pneumothorax occurrence (General FSVE, *p* = 0.214 and LAR E FSVE, *p* = 0.601), the results of the quantitatively recorded individual values indirectly reflect the degree of severity (MinIP value, *p* < 0.01 and LW value, *p* < 0.01). Satoh et al already concluded that MinIP imaging is more than 2½ times more predictive for the quantification of emphysema than HRCT [52]. In addition, our results suggest that for quantitative analysis, a comparison with normal lung parenchyma at the same level should be used rather than an exact value. It may, therefore, be possible to reduce the variability of the measured values and compensate for differences in X-ray beam energies [43] and location-related variations in radiodensity [15, 44, 53]. The superiority of MinIP images in our results suggests that these images identify earlier parenchymal alterations, which are also associated with a higher risk of pneumothorax. Consequently, according to the subgroup analysis, BROM-OLB MinIP was most reliable in patients with emphysematous changes but still showed strong test values in the seemingly normal subgroup. This aligns with the reported capability of MinIP images by Ghonge et al [39]. Smit et al [54, 55] already hypothesized that peripheral airway inflammation leads to airway obstruction with a check valve phenomenon, causing air trapping and the development of pneumothorax. Zhang et al [37] expressed concern about ROI measurements in the LW, as vessels, fibrosis, or inflammatory exudate around the lesion may also increase lung density and thus act as confounders.

This hypothesis could explain our finding that higher density values along the access path, as measured in the LW, act as an independent protective factor against pulmonary hemorrhage (grade 2 or higher) (*p* = 0.025). This observation is consistent with the study by Lee et al [19], which found that perilesional emphysema was significantly associated with the grade of hemorrhage (*p* < 0.01). Therefore, components of the lung scaffold structure are predominantly measurable in the LW rather than in MinIP images. Tai et al [16] suggested that this indirect reflection of the absence of lung scaffold structure was linked to the probability of hemorrhage. They hypothesized that destroying air-space walls distal to the terminal bronchioles may create increased potential space for hemorrhage to expand. Conversely, a compacted lung scaffold should have a protective effect. Future studies should investigate this directly by comparing destroyed lung parenchyma and edematous compacted lung parenchyma regarding the occurrence of pulmonary bleeding after biopsy.

Like Khan et al [3], we found a significantly higher incidence of pneumothorax with longer intervention times (*p* = 0.041), although we found no evidence of an independent association (*p* = 0.525). This is understandable for us, as many confounders influence the procedure time, such as patient compliance, access route length, and positioning [26] or the time the needle spends in the lung parenchyma [56].

Our study has several limitations. First, this was a retrospective analysis of a single center. Secondly, the placement of some ROIs is dependent on the radiologist’s experience, particularly in selecting inconspicuous lung parenchyma. In particular, our BROM-OLB model and its ROI measurements should be applied to 5 mm reconstructed slices to investigate the influence of noise. However, we believe that by focusing on the relative density differences between the ROIs in our model, the increased impact of noise in 1 mm slices is minimized. Additionally, the involvement of four different interventionalists may have influenced the complication rate. Therefore, our results should be externally validated to ensure their reliability. Third, due to technical limitations, we excluded a significant number of lesions directly adjacent to the pleura (< 10 mm) and inflammatory lung parenchyma in the access route. In future studies, a direct comparison of these factors should be conducted.

## Conclusion

The assessment of MinIP images in combination with relative quantitative measurement of radiodensity for access route planning can reduce the risk of pneumothorax in CT-guided lung biopsies. Our optimized, easy-to-use BROM-OLB measurement method, which requires no additional software, seems capable of detecting high-risk lung parenchymal changes associated with pneumothorax occurrence. Furthermore, it shows strong potential as a screening test for identifying patients at higher risk of pneumothorax, particularly in those with general emphysema or emphysema at the level of the access route.

## Supplementary information

ELECTRONIC SUPPLEMENTARY MATERIAL

## Data Availability

The datasets used and/or analyzed during the current study are available from the corresponding author upon reasonable request.

## Abbreviations

AUC: Area under the curve
BROM-OLB: Bridged radiological observations with measurement-optimized model
COPD: Chronic obstructive pulmonary disease
CT: Computed tomography
FSVE: Fleischner society visual emphysema CT pattern
HU: Hounsfield units
LW: Lung window
MinIP: Minimum-intensity projection
ROI: Regions of interest
