# Supplementary material for: Enhancing safety in CT-guided lung biopsies: correlation of MinIP imaging with pneumothorax risk prediction
Source: Insights Imaging. 2025 Jan 13;16:16. doi: 10.1186/s13244-024-01890-7 (PMC11730046; doi:10.1186/s13244-024-01890-7)
Supplement: Supplementary file 1 — ELECTRONIC SUPPLEMENTARY MATERIAL [file 13244_2024_1890_MOESM1_ESM.pdf]

Enhancing Safety in CT-Guided Lung Biopsies: Correlation of MinIP Imaging with Pneumothorax Risk Prediction

ELECTRONIC SUPPLEMENTARY MATERIAL

Fig. S1. Histological findings of the lung biopsies.

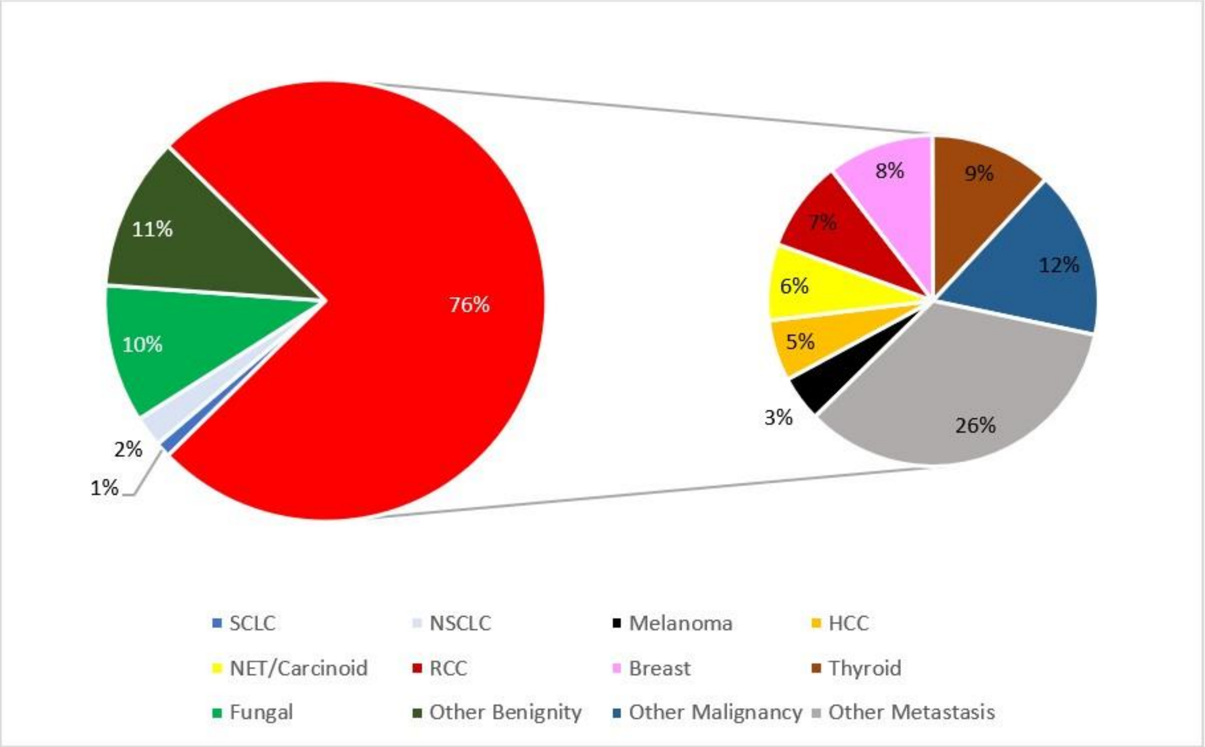

SCLC = small cell lung cancer; NSCLC= non small cell lung cancer; HCC = hepatocellular carcinoma; NET = neuroendocrine tumor; RCC = renal cell carcinoma.

Fig.S2. Age Distribution of Complications

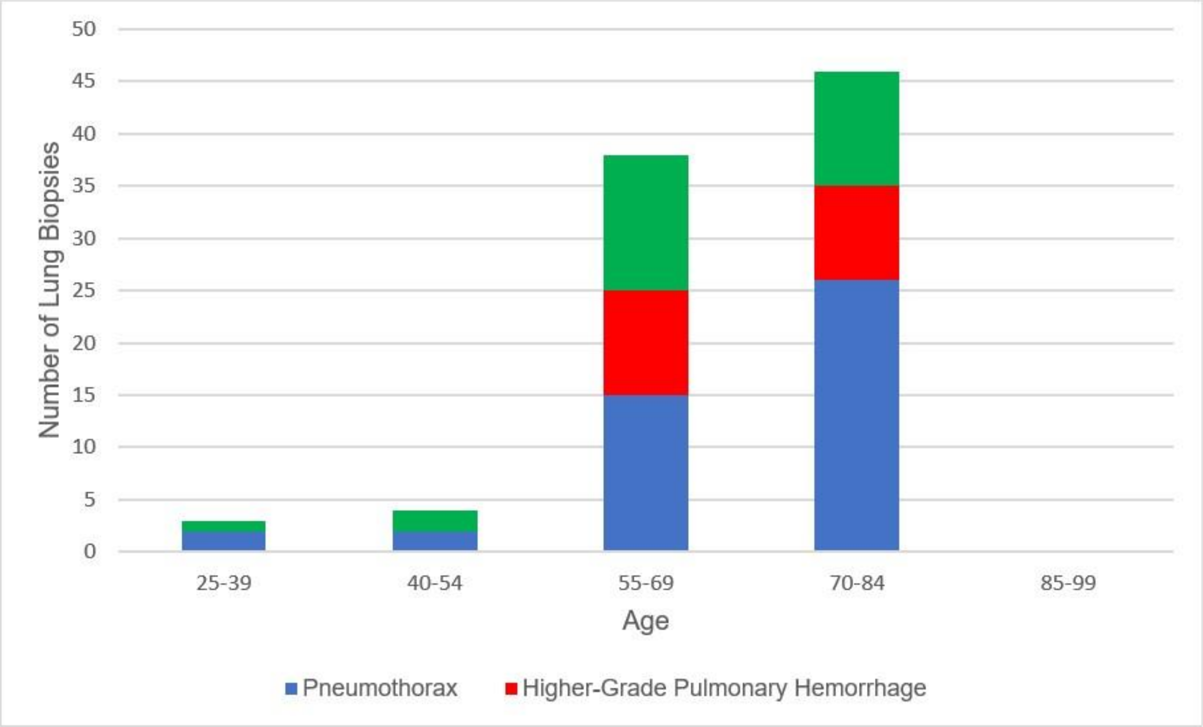

Table S1. Performance Metrics of Measurement Methods to Predict Pneumothorax.

Measuring Method	AUC	Sensitivity	Specificity	False-negative	False-positive
BROM OLB MinIP positive	0.845	81.80%	87.20%	18.2% (6/33)	12.8% (5/39)
BROM OLB LW positive	0.800	72.70%	87.20%	27.3% (9/33)	12.8% (5/39)
MinIP -868 HU	0.781	81.80%	74.40%	18.2% (6/33)	25.6% (10/39)
LW -769 HU	0.725	75.80%	69.20%	24.2% (8/33)	30.8% (12/39)
MinIP -850 HU positive	0.717	81.80%	61.50%	18.2% (6/33)	38.5% (15/39)
LW -850 HU positive	0.654	33.30%	97.40%	66.7% (22/33)	2.6% (1/39)

BROM-OLB = Optimised model for the relative quantitative measurement of radio density in CT-guided Lung biopsies; MinIP = Minimum Intesnity Projection; LW lung window; HU = Hounsfield Units; LAR E = Emphysema at the Level of Access Route; AUC = Area under the Curve.
